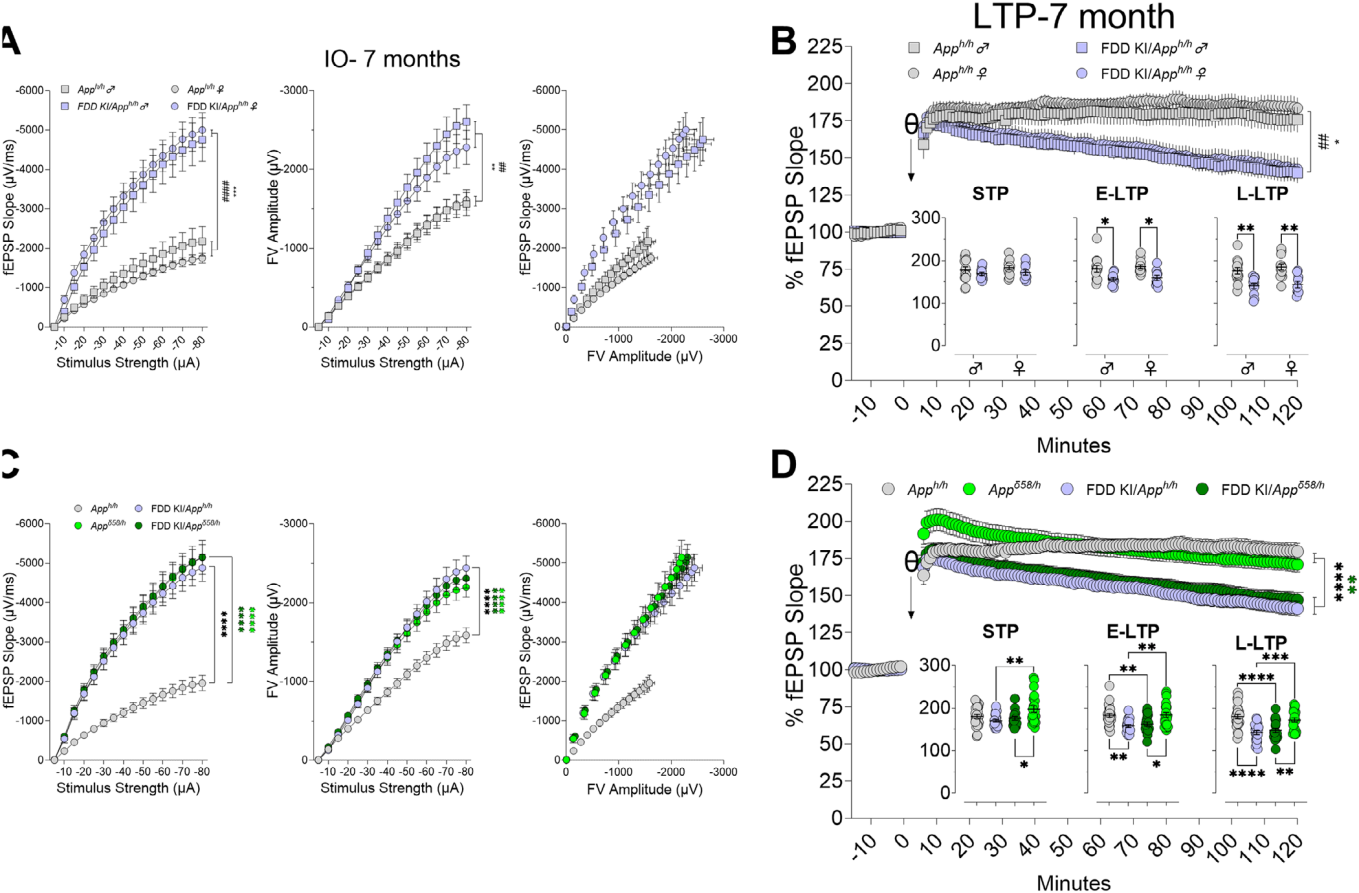# Synaptic Alterations in Familial Danish Dementia Knock‐In Rats

**DOI:** 10.1002/alz70855_098918

**Published:** 2025-12-23

**Authors:** Metin Yesiltepe, Luciano D'Adamio

**Affiliations:** ^1^ Rutgers Biomedical and Health Sciences, Newark, NJ, USA

## Abstract

**Background:**

Familial Danish Dementia (FDD) patients carry a heterozygous *Itm2b* mutation, which has been modeled in the FDD knock‐in (KI) rat to investigate synaptic alterations over time. Previous studies have shown increased basal synaptic transmission (BST) in young adult FDD‐KI rats; however, there is limited information regarding long‐term potentiation (LTP) changes with age and how the presence of the *App δ58* allele influences synaptic function.

**Method:**

To evaluate age‐related synaptic changes, we performed BST and LTP recordings in hippocampal slices from 7‐month‐old *FDD‐KI/App^h/h^* rats of both sexes. Additionally, we examined the effects of the *App δ58* allele by crossing FDD‐KI rats with *App^δ58/h^* rats and conducting electrophysiological recordings.

**Result:**

BST was significantly increased in 7‐month‐old *FDD‐KI/App^h/h^* rats compared to controls, consistent with findings in younger rats. However, the fiber volley (FV) amplitude was elevated at 7 months, suggesting increased afferent activation, which was not observed in younger rats. LTP was significantly impaired in 7‐month‐old *FDD‐KI/App^h/h^* rats, with reductions in both early (E‐LTP) and late phases (L‐LTP), while short‐term potentiation (STP) remained unaffected. In *FDD‐KI/App^δ58/h^* rats, BST and FV amplitude were similarly increased, but LTP impairments persisted, indicating that the *App δ58* allele does not rescue synaptic deficits.

**Conclusion:**

Our findings demonstrate that synaptic excitability increases with age in FDD‐KI rats, accompanied by progressive LTP impairments. The presence of the *App δ58* allele does not mitigate LTP deficits, highlighting the persistent impact of the *Itm2b* mutation on hippocampal function in the FDD model. These findings provide new insights into the synaptic changes associated with aging in FDD, addressing the previously unexplored LTP alterations.